# tDR-quant: a reliable electroporation-based approach for quantifying tRNA-derived fragments binding to ribosomes

**DOI:** 10.1093/femsyr/foaf051

**Published:** 2025-09-16

**Authors:** Kamilla Bąkowska-Żywicka, Agata Tyczewska

**Affiliations:** Institute of Bioorganic Chemistry Polish Academy of Sciences, Noskowskiego St. 12/14, 61-704 Poznań, Poland; Institute of Bioorganic Chemistry Polish Academy of Sciences, Noskowskiego St. 12/14, 61-704 Poznań, Poland

**Keywords:** *Saccharomyces cerevisiae*, tRNA-derived fragments, ribosome-associated noncoding RNAs, protein biosynthesis regulation, abiotic stress, ribosome

## Abstract

Ribosome-associated noncoding RNAs, particularly tRNA-derived fragments (tDRs), have emerged as key regulators of translation, especially under stress conditions. In *Saccharomyces cerevisiae*, tDRs interact with small ribosomal subunits to modulate protein biosynthesis, yet methods to quantitatively assess these interactions have been lacking. Here, we present tDR-quant, a robust technique for *in vivo* quantification of tDR/ribosome associations using electroporation of radiolabeled tDRs into yeast spheroplasts, followed by polysome profiling and radioactivity detection. We show that tDR interactions with ribosomes are stress- and dose-dependent, primarily associating with the 40S subunit but also with 60S, monosomes, and polysomes under specific conditions. Translation assays revealed that increased tDR levels inhibit protein synthesis without altering polysome profiles. Northern blot and quantitative real-time PCR (qRT-PCR) validated tDR-quant results, confirming its reliability. Stress-specific association patterns suggest that tDRs dynamically regulate translation by interacting with different ribosomal components in response to environmental cues. Importantly, these interactions do not correlate directly with tDR abundance, indicating selective ribosome binding. This study provides the first comprehensive method to quantify tDR–ribosome interactions *in vivo* and demonstrates that tDRs act as regulatory elements fine-tuning translation during cellular stress in yeast.

## Teaser

Overview of the tDR-quant protocol. The steps involve: (i) *S. cerevisiae* cell wall lysis with zymolyase, (ii) introduction of [^32^P]-labelled tDR to yeast spheroplasts, (iii) spheroplasts lysis, (iv) loading the cleared lysate on the sucrose gradient, (v) sucrose gradient ultracentrifugation and (vi) fractionation, and (vii) radioactivity measurements in collected fractions and profiling of noncoding RNAs–tDR accumulation. See the text for details.

## Introduction

Ribosomes are complex molecular machines responsible for protein biosynthesis in all living organisms. This process requires the involvement of a vast array of regulating factors that undergo many variations during each translation step. In 2012, it was discovered that a variety of small noncoding RNAs are associated with *Saccharomyces cerevisiae* ribosomes (Zywicki et al. 2012), indicating their participation in translation regulation. These RNAs represent a family of ribosome-associated noncoding RNAs (rancRNAs). Since that time, many classes of rancRNAs have been identified, including those derived from tRNAs [tRNA-derived fragments (tDRs)], snoRNAs [snoRNA-derived fragments (sdRNAs)], mRNAs, or rRNAs [reviewed in Rosina et al. (2025)].

The first described regulatory rancRNA in *S. cerevisiae* was an 18-nucleotide ncRNA derived from mRNA encoding tRNA methyltransferase TRM10 (Pircher et al. 2014). It was shown that the interaction of this rancRNA with the ribosome occurs under high salinity stress and causes a slowdown in the translation process through reduced affinity of tRNAs to the A site in the ribosome, and impaired structural dynamics of the entire ribosome (Reuther et al. 2021). Another striking discovery on rancRNAs was made in *Escherichia coli*, where the processing of mature 16S rRNA to a ∼80-nt-long fragment was revealed (Luidalepp et al. 2016). This rRNA fragment was generated solely during the stationary growth phase and remained attached to the 30S small ribosomal subunit, evoking reduced rates of protein biosynthesis.

In later studies using *S. cerevisiae*, we have demonstrated that the occurrence in the cytoplasm and the interaction of ranc–sdRNAs with ribosomes in *S. cerevisiae* are stress-dependent and cause functional consequences in the form of reduced translational activity of yeast under stress (Mleczko et al. 2019). Moreover, we have shown that the association of ranc–tDRs with *S. cerevisiae* ribosomes is tightly regulated by diverse stress conditions (Bąkowska-Żywicka et al. 2016a). The site of interaction of yeast tDRs with the ribosomes was defined as a small ribosomal subunit, similarly to ranc–16SrRNA-derived RNA in *E. coli*. Strikingly, ranc–tDRs in halophilic archaeon *Haloferax volcanii* were also shown to bind to the small ribosomal subunit (Gebetsberger et al. 2017).

tDRs are now recognized as a broad and functionally diverse class of small RNAs processed from mature or precursor tRNAs. Two types of tDRs can be distinguished: longer tRNA halves, typically 30–35 nts, and shorter fragments of 14–26 nts, which can originate from either 5′ or 3′ ends or the internal regions of mature tRNAs. In addition, tDRs can be generated from the 3′ trailer sequences of pre-tRNAs in the nucleus, as demonstrated in human cell lines following infection with respiratory syncytial virus (Choi et al. 2021). The biogenesis of tDRs relies on endonucleolytic cleavage carried out by various RNases. In humans, RNase A family enzyme angiogenin is a key contributor (Yamasaki et al. 2009), whereas in *S. cerevisiae*, the RNase T2 family member Rny1p mediates cleavage (Thompson and Parker 2009). Additional enzymes, including SLFN11, SLFN13, and RNase L have also been implicated in the production of cytosolic tDRs in human cells (Donovan et al. 2017, Li et al. 2018, Yang et al. 2018).

tDR biogenesis is often stress-induced and condition-dependent, which allows them to act as rapid regulators of translation, acting through both base-pairing-dependent and -independent mechanisms. Sobala and Hutvagner (2013) reported that 5′ tDRs repress translation without engaging in base-pairing, instead associating with polysomes to exert their regulatory effect. Their findings highlighted the importance of a GG dinucleotide motif, necessary but not sufficient, for inhibiting translation. In archaea, tDRs which bind to the small ribosomal subunit repress peptide bond formation, directly inhibiting translation (Gebetsberger et al. 2017). Similarly, in mammalian cells, tDRs were shown to associate with ribosomes or initiation complexes, displacing factors such as eIF4G/A and PABPC1, resulting in global translational repression (Ivanov et al. 2011). A specific structural motif, the 5′ terminal oligo-guanine, consisting of four or five consecutive guanines, was identified as a critical feature for this inhibitory effect. This follow-up study was inspired by previous observations that stress-induced by arsenite treatment, heat shock, and UV radiation cleavage of tRNAs in U2OS cells *via* angiogenine leads to translation inhibition in a phospho-eIF2⍺ independent manner (Yamasaki et al. 2009). In human 293 and HCT116 cells (Haussecker et al. 2010, Kuscu et al. 2018), and B lymphoma cells (Maute et al. 2013) tDRs regulate gene expression *via* a miRNA-like pathway through interactions with Argonaute proteins. Importantly, yeast tDRs contribute to the regulation of protein biosynthesis primarily by associating with ribosomes and aminoacyl-tRNA synthetases to inhibit translation, particularly under stress conditions (Tyczewska and Grzywacz 2023). This regulatory mechanism allows cells to fine-tune protein production in response to environmental changes. *Saccharomyces cerevisiae* tDRs affect aminoacylation of the corresponding parental tRNAs, as well as global aminoacylation through forming a ternary complex tDR/ribosome/aminoacyl-tRNA synthetase (Mleczko et al. 2018). Collectively, all of the above studies indicate that tDRs regulate protein biosynthesis both through ribosome interaction and through targeting specific mRNA translation pathways.

Nowadays, it is undeniable that tRNAs are processed to tDRs in a huge variety of organisms, that tDRs play crucial roles in protein biosynthesis, and that their function is induced or elevated by environmental stresses. However, there is still limited data on tDR/ribosome association dynamics, especially during stress. Therefore, we have noticed an urgent need for establishing a reliable method of quantifying tDR/ribosome interactions in *S. cerevisiae*, an organism where tDRs function and association with small ribosomal subunits were described. Here we present reliable and straightforward method for quantitation of tDR/ribosome interactions directly in the yeast cells, based on electroporation of labeled small RNAs into live yeast cells and verification of interactions *via* polysome profiling combined with radioactivity monitoring (tDR-quant). The utility of the method was further validated with well-established methods of small RNA detection, namely northern blot and quantitative real-time PCR. Using a newly established method, we provide a first comprehensive analysis of tDRs' association with yeast ribosomal components, depending on the tDR amount in the yeast cells. Moreover, we provide evidence that tDR/ribosome interactions are stress-dependent and that yeast tDRs associate primarily with small ribosomal subunits in all growth conditions.

## Materials and methods

### tDR-quant protocol overview

Yeast spheroplasts (yeast cells deprived of the cell wall and therefore competent for DNA or RNA uptake) were prepared using zymolyase, combined with 5‐[^32^P]‐end‐labelled synthetic tDR, and electroporated. Then, spheroplasts were lysed and loaded on top of an 8%–40% sucrose gradient. After ultracentrifugation, the gradient was monitored at A_254_ using a flow cell coupled to a spectrophotometer and then fractionated into equal fractions: free RNAs (top fraction) were separated from ribosome-associated RNAs (bottom fractions, including 40S-associated RNAs, 60S-associated RNAs, monosome-associated RNAs, and polysome-associated RNAs). Fractions were then processed for radioactivity quantification with the scintillation counter, and the accumulation of tDRs was analyzed in each fraction. A schematic overview of the tDR-quant protocol is presented in the Graphical abstract.

### Detailed protocols

#### tRNA-derived fragments

tDRs for this analysis have been chosen based on our previous screens for ribosome-associated rancRNAs (Zywicki et al. 2012), followed by studies on their influence on protein biosynthesis (Bąkowska-Żywicka et al. 2016a, Mleczko et al. 2018). The four tDRs we focused on had robust read coverages of over 40 reads in the rancRNA library, and possessed a strong inhibitory effect on translation in *S. cerevisiae*. Two of them were derived from tRNA–His–GTG (5′ as well as 3′-end), and the other two from the 3′-part of tRNA–Ser–AGA. RNA oligonucleotides were chemically synthesized. Sequences of the oligonucleotides are presented in Table 1.

**Table 1.** Oligonucleotides used in this study.

Name	Type	Sequence (5′-3′)	Length (nt)
tDR-1:29-His–GTG	**RNA**	**GCCAUCUUAGUAUAGUGGUUAGUACACAU**	**29**
	RT primer	GTCGTATCCAGTGCAGGGTCCGAGGTATTCGCACTGGATACGACATGTGT	50
	Fwd primer	GCGGCGGCCATCTTAGTATAGTGGTTAGT	23
	asDNA	TGTGTACTAACCACTATACTAAGATGG	29
tDR-40:67-His–GTG	**RNA**	**GAUGAAACCCUGGUUCGAUUCUAGGAGAUGG**	**27**
	RT primer	GTCGTATCCAGTGCAGGGTCCGAGGTATTCGCACTGGATACGACCCATCT	50
	Fwd primer	GCGGATGAAACCCTGGTTCGATTCTAGG	28
	asDNA	AGAATCGAACCAGGGTTTCA	20
tDR-62:82-Ser–AGA	**RNA**	**GGUUCGAGUCCUGCAGUUGU**	**20**
	RT primer	GTCGTATCCAGTGCAGGGTCCGAGGTATTCGCACTGGATACGACACAACT	50
	Fwd primer	GCGGCGGGGTTCGAGTCCTGC	21
	asDNA	ACAACTGCAGGACTCGAACC	20
tDR-58:82-Ser–AGA	**RNA**	**CGCAGGUUCGAGUCCUGCAGUUGU**	**24**
	RT primer	GTCGTATCCAGTGCAGGGTCCGAGGTATTCGCACTGGATACGACACAACT	50
	Fwd primer	GCGGCGGCGCAGGTTCGAGTCCTGC	25
	asDNA	ACAACTGCAGGACTCGAACC	21
Control	**RNA**	**AUAGGCCAUAAGGAGUCUCGGUACGUCUUGUAUG**	**44**
	RT primer	GTCGTATCCAGTGCAGGGTCCGAGGTATTCGCACTGGATACGACCATACA	50
	Uni primer	CCAGTGCAGGGTCCGAGGTA	20

tDR sequences are presented in bold. RT primer, stem-loop primer used for reverse transcription; Fwd primer, forward PCR primer; Uni primer, universal reverse PCR primer; asDNA, northern blot probe.

A 44-nucleotide long control oligomer has been designed to reflect the mean nucleotide composition of all tested tDRs. This is a synthetic sequence that does not share significant homology with known cellular tRNAs, tDRs or sequences involved in ribosome association. It was originally designed to serve as a nonfunctional, size-comparable control for RNA uptake and stability analyses. While we acknowledge that it is longer than tested tDRs, we chose this length to avoid potential interactions with ribosomal particles.

#### Strain and growth conditions

*Saccharomyces cerevisiae* wild-type strain BY4741 (MATα; his3Δ1; leu2Δ0; met15Δ0; ura3Δ0) was used in all assays. The cultures were initiated by inoculating frozen stock cultures onto solid YPD plates (1% yeast extract, 2% peptone, 2% glucose, and 2% agar). Then, single colonies from YPD plates were inoculated onto YPD liquid medium and grown at 30°C with continuous agitation at 180 rpm. Cells were grown in optimal conditions for tDR-quant and in 12 different growth conditions for northern blot and qRT-PCR assays, as previously described (Pietras et al. 2025). The duration of stress was based on previous data reported by Causton et al. (2001), who tested 15, 30, 45, 60, and 120 min of stress and reported that the exposure to 15-min stress alters the expression of 66% of *S. cerevisiae* genes. Moreover, in our own studies, after 15-min stress, we have observed prominent changes in processing of tDRs (Bąkowska-Żywicka et al. 2016b), ribosome/small RNAs interactions (Zywicki et al. 2012), the composition of the ribosomes (Pietras et al. 2024), as well as growth, fitness, and single-cell morphology changes (Pietras et al. 2025). Briefly, cells were grown to mid-log phase (OD_600_ of 0.8), and stress conditions were applied for 15 min. The temperature shifts to 37°C (heat shock) or to 15°C (cold shock) were carried out by the addition of an equal volume of YPD prewarmed to 50°C or prechilled to 4°C, respectively. The heat‐shocked cultures were continued to grow for 15 min at 37°C, and cold‐shocked at 15°C. The cultures were either supplemented with 1 M NaCl (high salt conditions), with 0.1 M Tris–HCl pH 8.3, resulting in a final pH of 7.9 (high pH conditions), or with 1 M citric acid (low pH conditions of pH 4.0). UV exposure was performed in a Stratalinker (Stratagene, La Jolla, CA). Cells were grown to mid‐log phase, then moved into Petri plates and exposed to 120 J/m^2^ UV for 30 s. Yeast were returned to a flask and continued to grow for further 15 min. To induce hyperosmotic shock, the cultures were supplemented with 1 M sorbitol. For hypoosmotic conditions, the cells were grown to mid‐log phase in YPD supplemented with 1 M sorbitol, then collected by centrifugation, and resuspended in YPD without sorbitol. For amino acid and sugar starvation stresses, cells were collected by centrifugation at mid‐log phase, washed in starvation medium, and further grown in medium lacking amino acids or sugar, respectively. In parallel, anaerobic and normal growth of *S. cerevisiae* was performed. Subsequently, 15 min before harvest, 500 µg/ml cycloheximide (f.c.) was added to the culture.

#### Spheroplast preparation and electroporation

Yeast spheroplasts were prepared from a 50 ml culture grown to an OD_600_ of 0.8 by adding 350 U of zymolyase (Zymo Research) and incubating at 30°C for 25–30 min as previously described (Mleczko et al. 2018). Spheroplasts (115 µl) were combined with 1–50 pmol synthetic 5‐[^32^P]‐end‐labeled tDRs or control RNA and electroporated (1,500 V, 25 μF, and 200 Ω) using a BioRad Micro Pulser. Subsequent to electroporation, 1 ml YPD supplemented with 1 M sorbitol was added to the spheroplasts, transferred into a 1.5 ml Eppendorf tube, and incubated at 30°C for 45 min before adding cycloheximide (500 µg/ml) and incubating for an additional 15 min.

#### Polysome profiling

Polysome profiling experiments were conducted as previously described (Mleczko et al. 2018). For tDR-quant, yeast spheroplasts were lysed with repetitive shearing through the 20 G needle. For northern blot and qRT-PCR assays, cell lysate was prepared by vortexing yeast cells with glass beads (600 nm) and with RNAse inhibitor. Lysates were purified by centrifugation at 11,300  × *g* for 2 min at 4°C and at 11 300  × *g* for 10 min at 4°C. Approximately 30 A_260_ units of cell lysates were loaded onto a linear 8%–40% sucrose gradients. The gradients were centrifuged in a Beckman SW40 Ti rotor at 39,000 rpm for 2.5 h at 4°C. Sucrose gradients were analyzed by continuously monitoring absorbance at A_254_ with a UV detector. Fractions of 33 droplets were automatically collected and subjected to liquid scintillation counting (tDR-quant) or to RNA isolation (northern blot and qRT-PCR assays). Polysome profiling experiments were performed at least in triplicate.

#### Translation assays

Yeast ribosomes and soluble protein factors for *in vitro* translation assays were prepared as described (Bąkowska-Żywicka et al. 2016a). Briefly, cell cultures were grown at 30°C to early log phase. Sodium azide (1 mM NaN_3_) was added to the culture 15 min before the harvest. Cell pellets were washed with water and resuspended in 1 ml of buffer A (10 mM MgCl_2_, 100 mM KCl, 50 mM Tris/HCl, pH 7.5, 0.4 mM PMSF) at 4°C. An equal volume of acid-washed glass beads (400 µm in diameter) was added, and cells were broken by 10–15 pulses of vortexing (15 s each), punctuated with cooling on ice. Cell debris was precipitated at 27,000 × *g* for 15 min at 4°C. Lysate was clarified by centrifugation at 30,000 × *g* for 20 min at 4°C. After clarification, the ribosomes were pelleted from the lysates by centrifugation at 160,000 × g for 90 min at 4°C. Pelleted ribosomes were resuspended in buffer B (2 mM MgAc_2_, 100 mM KAc, 20 mM HEPES/KOH pH 7.4, 0.1 mM PMSF, 1 mM DTT, 20% glycerol) and stored at −80°C. The remaining supernatant, containing soluble protein factors, was aliquoted and stored at −80°C.

*In vitro* translation assay was carried out as described (Bąkowska-Żywicka et al. 2016a) using 5 A_260_ units of the yeast ribosomes, 25 mg poly(U), 100 mg of soluble protein factors, 25 mg deacylated yeast tRNA, and 0.3 nmol (^3^H)-phenylalanine. The reaction was performed at 30°C for 30 min. Labeled proteins were precipitated by adding 1 ml of 20% (f.c.) trichloroacetic acid and incubating at 95°C for 20 min. This solution was then filtered through a glass-fiber filter and quantified by liquid scintillation counting. The measurements were performed at least in triplicate.

*In vivo* translation assay (metabolic labeling) was performed as previously described in Mleczko et al. (2018). Yeast spheroplasts (115 µl) were combined with 1–50 pmol synthetic tDRs and electroporated (1,500 V, 25 *µ*F, and 200 Ω) using a BioRad Micro Pulser. For a control, translation was inhibited by adding 7.5 µg/µl cycloheximide to the spheroplasts. Subsequent to electroporation, 1 ml YPD supplemented with 1 M sorbitol was added to the spheroplasts, and the reaction was transferred into a 1.5 ml Eppendorf tube. Next, 450 µl of the sample was incubated at 30°C for 15 min before adding 1 µl ^35^S-methionine (1,000 Ci/mmol, 10 mCi/ml) and incubating for an additional 1 h. Labeled proteins were precipitated by adding 1 ml of 20% (f.c.) trichloroacetic acid and incubating at 95°C for 20 min. This solution was then filtered through a glass-fiber filter and quantified by liquid scintillation counting. Metabolic labeling measurements were performed at least in triplicate.

#### RNA isolation

RNA was isolated with TRIzol Reagent according to the manufacturer’s protocol with a few changes. Briefly, 500 µl of TRIzol was added to each fraction, probes were mixed and frozen in liquid nitrogen. After slow defrosting on ice, 100 µl of chloroform was added to each probe, mixed for 15 s, and centrifuged at 12,000  × *g* for 15 min at 4°C. RNA was precipitated and assessed for quality and quantity.

#### Northern blot analysis

RNA isolated from polysome profiling fractions was separated on 12% denaturing polyacrylamide gels and electrotransferred to Amersham Hybond N + membranes as previously described (Bąkowska-Żywicka et al. 2016b). Nucleic acids were UV‐cross‐linked to the membranes. The 5‐[^32^P]‐end‐labeled DNA oligonucleotides were used as antisense probes (Table 1). Hybridization was carried out overnight, and two‐step washing was performed afterwards. The membranes were exposed on the phosphor–storage intensity screen (Fujifilm) overnight. Screens were scanned with Fujifilm Fluorescent Image Analyzer FLA–5100 and analyzed quantitatively with the densitometric program Multi-Gauge Image Analyzer. Northern blot assays were performed at least in duplicate.

#### Quantitative real-time PCR

Reverse transcription reactions using stem-loop primers for tDR detection were performed as described in Mleczko et al. (2019), using 10 ng RNA from polysome profile fractions or total RNA, 50 nM of each stem-loop RT primer for tDRs and spike-in RNA, 1  ×  RT buffer, 0.25 mM of each dNTP, 50 U SuperScript SSII reverse transcriptase (Invitrogen), 5 U RiboLock RNase Inhibitor (Thermo Scientific), 10 mM DTT, and 500 fM spike-in control RNA (Table 1) as a normalizer. Twenty-microlitre reactions were incubated in a Bio-Rad T100TM Thermocycler for 30 min at 16°C, followed by pulsed RT of 60 cycles at 30°C for 30 s, 42°C for 30 s, and 50 °C for 1 s. Quantitative real-time PCR assays were performed in triplicate.

## Results

**Figure 1.**
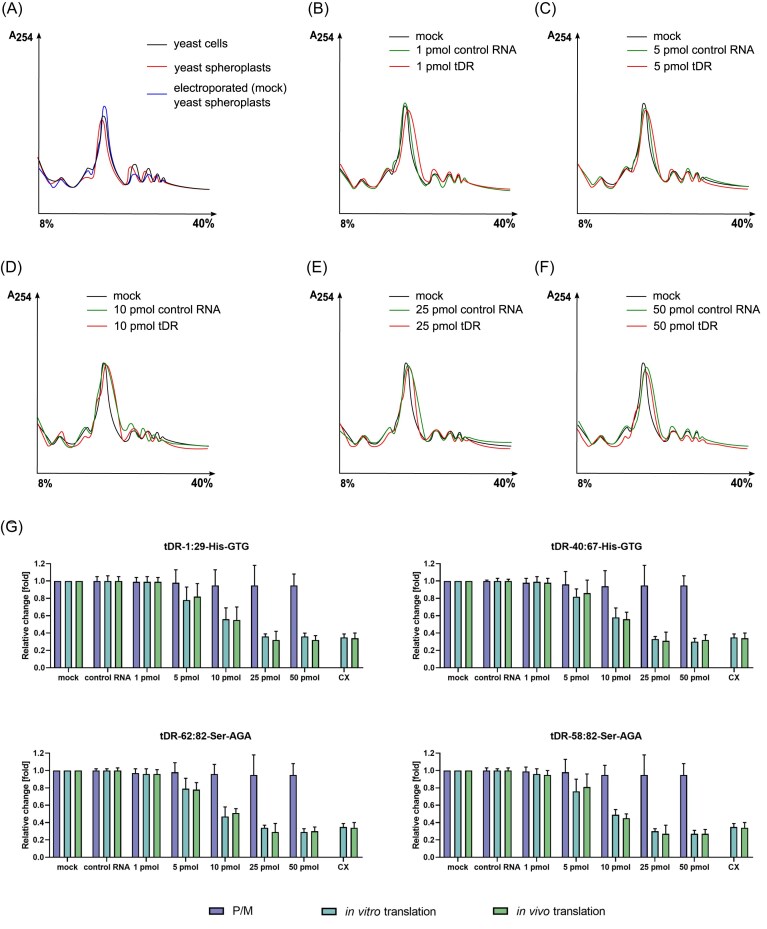
Translational activity of yeast spheroplasts. (A) A comparison of polysome profiles derived from intact yeast cells, yeast spheroplasts, and spheroplasts electroporated with water. (B–F) A comparison of polysome profiles of yeast spheroplasts electroporated with 1–50 pmol of tDR-1:29-His–GTG or 1–50 pmol of control RNA. (G) P/M ratio, in vitro translation [yeast poly(Phe) synthesis in the isolated *in vitro* translation system], and in vivo translation (incorporation of ^35^S-methionine into the translatome of yeast spheroplasts) results in relation to control conditions (no RNA, mock) are presented. P/M and the efficiency of translation in control conditions were set as 1.0, and the results obtained in yeast spheroplasts electroporated with 1–50 pmol of tDR or 50 pmol of control RNA were compared to this value. CX, cycloheximide.

At the beginning, we have optimized the amount of RNA electroporated into yeast spheroplasts. We assumed that in order to assess actual, physiological interactions of tDRs with particular components of the ribosome, the polysome profiles should be intact. We have therefore inspected the polysome profiles of intact yeast cells, yeast spheroplasts, as well as electroporated spheroplasts (without the addition of exogenous RNA, Fig. 1A). It appeared that neither spheroplasting nor electroporation changed the polysome profiles significantly and all profiles showed an accumulation of multiple ribosomes bound to mRNAs (polysomes) toward the bottom (40%) of the gradient (Fig. 1B). These observations were further supported by the analysis of the polysome to monosome ratio (P/M), which remained unaltered after electroporation with increasing amounts of tDRs (Fig. 1C).

In the next step, we measured both *in vitro* and *in vivo* translational activity of yeast exposed to increasing amounts of tDRs. We employed two assays: (i) an *in vitro* system, where the ribosomes and proteins needed for translation were isolated from intact yeast cells, and tDRs were introduced directly to this system, and (ii) an *in vivo* system, where increasing amounts of tDRs were electroporated to yeast spheroplasts together with ^35^S-labelled methionine, and the incorporation of ^35^S-Met into the translatome of yeast spheroplasts was measured. We observed that despite no significant changes in polysome profiles, both *in vitro* and *in vivo* translational activity was severely impaired in the presence of yeast tDRs (Fig. 1C). Translation efficiency decreased gradually with increasing tDR dose, and the addition of 25 or 50 pmol of tDRs resulted in translation inhibition in a range similar to the well-known ribosome-targeting antibiotic cycloheximide.

These results clearly indicate that when tDRs are present in yeast in small doses (which most probably reflect optimal, physiological conditions), both polysome profiles and translation efficiency are unaltered. However, with increasing amounts of tDRs (which most probably reflect nonoptimal, stress conditions), translation efficiency drops down, despite unaltered polysome profiles. Our recent publication tackled the point of ribosomal heterogeneity during nonoptimal conditions of yeast growth and revealed that under stress conditions, the heterogeneity of the ribosomes may provide means to prepare the cells for quick recovery (Pietras et al. 2024).

**Figure 2.**
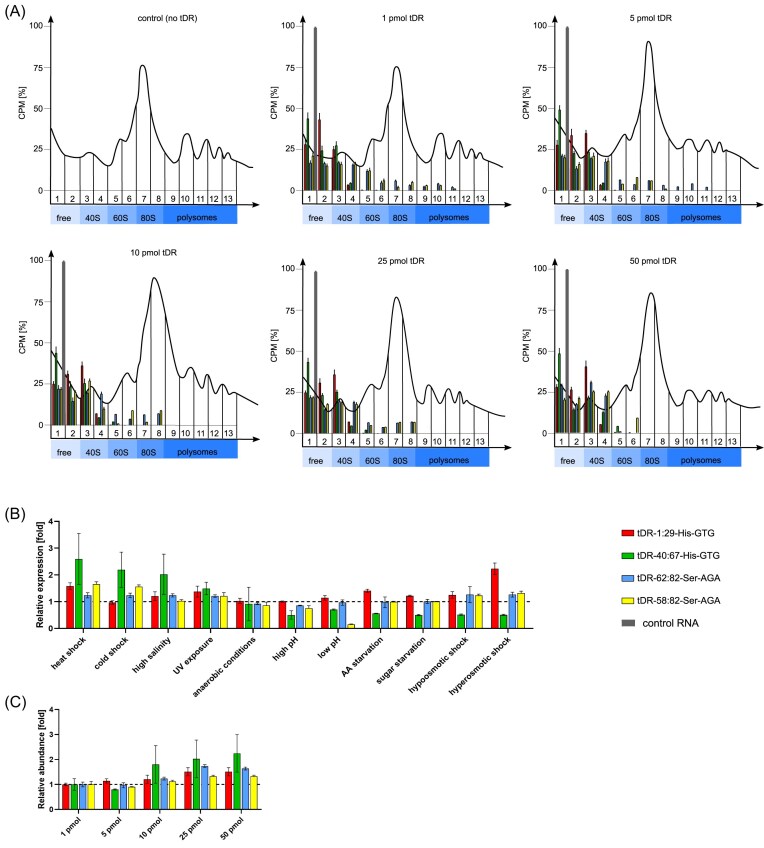
Polysome association and relative abundance of synthetic and endogenous tDRs in yeast. (A) Polysome profiles derived from yeast sphereplasts electroporated with 1–50 pmol of tDR or control RNA, as well as quantitation of radioactivity retained in each fraction, are presented. CPM% (counts per minute) was calculated as a percentage of CPM for a particular tDR in a selected fraction in relation to the sum of CPM for the particular tDR in all fractions. (B) Relative expression of endogenous tDRs measured with quantitative real-time PCR. The expression of endogenous tDRs was set to 1 under optimal yeast growth conditions. (C) Relative abundance of synthetic tDRs measured with tDR-quant. The abundance of tDRs was set to 1 under conditions of 1 pmol of electroporated tDR.

Taking into consideration that tDR presence did not change the polysome profiles but still affected translation efficiency *in vivo* and *in vitro*, we aimed at precise quantitation of tDR association with particular ribosomal fractions. We have therefore applied the tDR-quant technique: we have electroporated different amounts of 5‐[^32^P]‐end‐labeled tDRs to yeast spheroplasts, performed polysome profiling, collected 13 fractions per profile, and measured radioactivity level retained in each fraction (corresponding to a particular tDR). We observed that all four tested tDRs were present mainly in the upper fractions, corresponding to free RNAs and 40S-associated rancRNAs (Fig. 2A). Control RNA was present solely in the fractions corresponding to free, nonribosome-bound RNAs. Importantly, to assess the physiological relevance of synthetic tDR introduction, we quantified the amount of electroporated tDRs taken up by yeast spheroplasts with the means of tDR-quant (Fig. 2C) and compared it to the endogenous levels of the same tDRs in cells under optimal and stress conditions (with the means of real-time PCR, Fig. 2B). We normalized the abundance of synthetic tDRs to 1 when 1 pmol was introduced (Fig. 2C), and set the expression of endogenous tDRs to 1 under optimal yeast growth conditions (Fig. 2B). Our measurements showed that the relative abundance of synthetic tDRs remained around 1 when small amounts (5 pmol) were introduced via electroporation, and increased when higher amounts (10–50 pmol) were used. The observed fold-change ranged from 1.2 to 2.2. Similarly, endogenous tDR levels were upregulated under various stress conditions, including heat shock, cold shock, high salinity, and hyperosmotic stress, with increases within the same order of magnitude. These results suggest that the observed ribosomal associations are not merely due to artificial oversaturation but likely reflect physiologically relevant interactions that occur under stress-induced elevations of tDRs.

**Figure 3.**
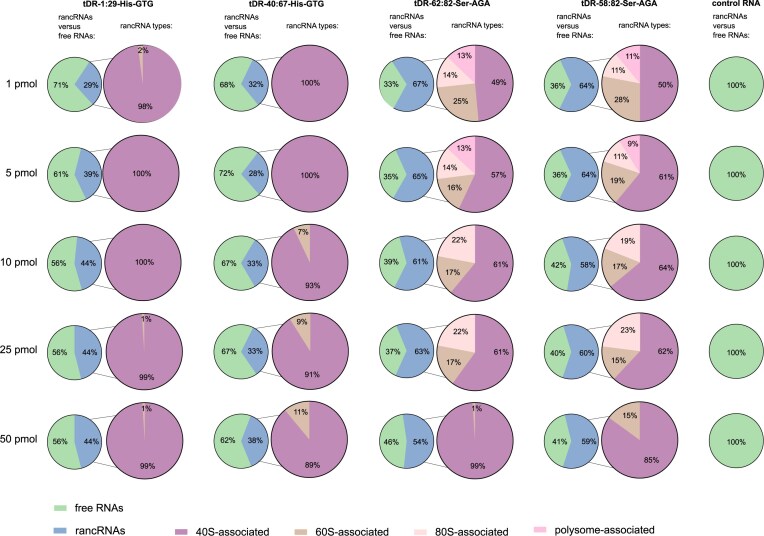
Dose-dependent association of tDRs with ribosomal particles in yeast spheroplasts. tDRs derived from tRNA–His–GTG (tDR-1:29 and tDR-40:67) and tRNA–Ser–AGA (tDR-62:82 and tDR-58:82) were electroporated into yeast spheroplasts at increasing concentrations (1–50 pmol), followed by polysome profiling and quantification of their co-migration with ribosomal subunits (40S, 60S), 80S monosomes, and polysomes. Part-of-the-whole graphs present the ratio of co-migration of particular tDRs with free RNA and rancRNA fractions.

In the next step, we have quantified the degree of co-migration of tDRs with ribosomal particles: small ribosomal 40S subunits, large ribosomal 60S subunits, 80S monosomes, and polysomes. We observed that both tDRs derived from tRNA–His–GTG were mostly present in the free RNAs fraction and both tDRs derived from tRNA–Ser–AGA were mostly rancRNAs (Fig. 3). Up to 44% of tDR-1:29-His–GTG and up to 38% of tDR-40:67-His–GTG were present in yeast spheroplasts as ribosome-bound, mostly as 40S-interacting rRNAs (99%–100% of ribosome-bound tDR-1:29-Hi–-GTG was associated with 40S subunits, and 89%–100% of ranc–tDR-40:67-His–GTG was associated with 40S subunits, depending on the amount of electroporated tDR). With increasing amounts of tDR-40:67-His–GTG (but not tDR-1:29-His–GTG), an observable shift was observed in favor of 60S subunits: when 1 pmol or 5 pmol of this tDR were introduced to yeast spheroplasts, tDR/40S subunit interactions were observed solely, but with 10, 25, or 50 pmol, 7%, 9%, or 11% of this tDR co-migrated with 60S subunits, respectively. This observation might imply that with increasing amounts of tDR-40:67-His–GTG, 40S subunits are the first to interact with; however, a portion of tDR is bound to 60S subunits as well. None of the tDRs derived from tRNA–His–GTG were observed as co-migrating with monosomes or polysomes.

Both tDRs derived from tRNA–Ser–AGA were also mostly associated with 40S subunits (49%–99% of ribosome-bound tDR-62:82-Ser–AGA was associated with 40S subunits, and 50%–85% of ranc–tDR-58:82-Ser–AGA was associated with 40S subunits, depending on the amount of electroporated tDR). However, prominent amounts of tRNA–Ser-derived tDRs were also co-migrating with 60S subunits (17%–25% of ranc–tDR-62:82-Ser–AGA and 15%–28% of ranc–tDR-58:82-Ser–AGA), and 80S monosomes (14%–22% of ranc–tDR-62:82-Ser–AGA and 11%–23% of ranc–tDR-58:82-Ser–AGA). Co-migration of tDRs with polysomes was observed only when lower amounts of tDRs were electroporated to yeast spheroplasts: 13% of ranc–tDR-62:82-Ser–AGA was associated with polysomes in spheroplasts, and 9% and 11% of ranc–tDR-58:82-Ser–AGA were associated with polysomes in spheroplasts when 1 or 5 pmol tDR were electroporated, respectively. Such dose-dependent dynamics of tDRs–Ser/ribosome interactions together with dose-dependent translation inhibition (Fig. 1C) might suggest that tDRs–Ser/polysome interactions are needed for proper efficiency of translation (100% and ∼80% translation efficiency when 13% of ranc–tDRs–Ser were interacting with polysomes after 1 or 5 pmol tDR electroporation, respectively), and mimic the optimal, physiological conditions of yeast cells, since it has been previously reported that particular tDR levels are at constant, lower than in nonoptimal conditions, levels in yeast cells (Bąkowska-Żywicka et al. 2016b). Addition of higher doses of 10 and 25 pmol of tDRs–Ser resulted in their co-migration with 40S and 60S subunits as well as the whole 80S ribosomes, and 50 pmol in co-migration primarily with 40S subunits. This situation strongly suggests that 40S is a primary target of tDRs when they are at doses potent to inhibit translation. This situation might also mimic the stressed yeast cells, where the selected tDR levels are upregulated and translation is altered (Bąkowska-Żywicka et al. 2016a).

Concluding the data presented on Fig. 3, it is a clear demonstration of a dose-dependent and tDR-specific pattern of interaction between tDRs and ribosomal particles in yeast spheroplasts. tDRs interact with ribosomes in a concentration-dependent and tDR-specific manner. At low concentrations, Ser-derived tDRs support translation via polysome interaction, but at higher levels, both His- and Ser-derived tDRs preferentially bind ribosomal subunits, correlating with translation inhibition and a stress-like cellular state.

Importantly, control RNA was present in free RNA fractions solely, independently of the amount electroporated to yeast spheroplasts.

In order to verify the reliability of the results obtained with the newly established tDR-quant method, we have employed two gold-standard techniques of small RNA detection, namely northern blotting and quantitative real-time PCR. We performed polysome profiling of yeast cells grown under optimal conditions, collected 13 fractions/profile, isolated RNA, and performed northern blots or qRT-PCRs.

**Figure 4.**
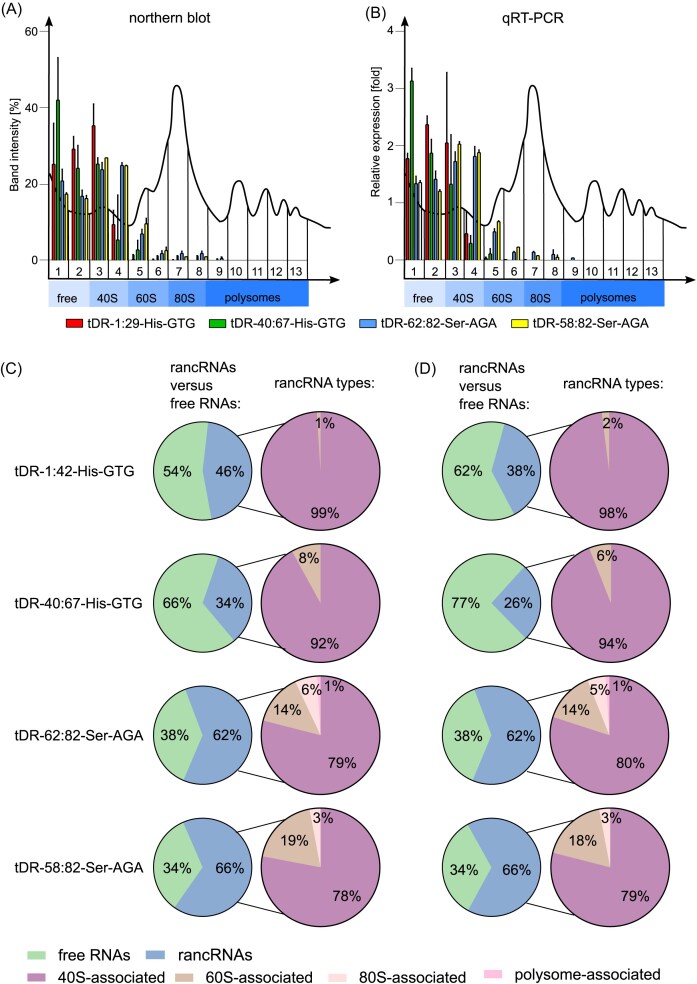
tDR-quant results verification with northern blot and quantitative real-time PCR. Polysome profiles derived from yeast, as well as quantitation of band intensity corresponding to tDR (A) or relative tDR expression (B) in each fraction, are presented. Graphical presentation of northern blot (C) and quantitative real-time PCR (D) results. Part-of-the-whole graphs present the ratio of co-migration of particular tDRs with free RNA and rancRNA fractions.

We observed that all four tested tDRs were present mostly in the upper fractions, corresponding to free RNAs (fractions 1 and 2) and 40S-associated rancRNAs (fractions 3 and 4, Fig. 4A, B), similarly to the results obtained using tDR-quant.

Both tDRs derived from tRNA–His–GTG were mostly present in the free RNA fraction (54% and 66% as measured with northern blot, and 62% and 77%, as measured with qRT-PCR, respectively, Fig. 4C, D), which is consistent with tDR-quant results. Both tDRs derived from tRNA–Ser–AGA were mostly rancRNAs (62% and 66% as measured with northern blot or qRT-PCR, respectively, Fig. 4C, D), which is also consistent with tDR-quant results. A total of 46% and 38% of tDR-1:29-His–GTG, and 34% and 26% of tDR-40:67-His–GTG were detected with northern blot and qRT-PCR, respectively, as ribosome-bound, mostly as 40S-interacting rancRNAs: 99% (northern blot) or 98% (qRT-PCR) of ranc–tDR-1:29-His–GTG was associated with 40S subunits, and 92% (northern blot) or 94% (qRT-PCR) of ranc–tDR-40:67-His–GTG was associated with 40S subunits. None of the tDRs derived from tRNA–His–GTG were observed as co-migrating with monosomes or polysomes, as observed in tDR-quant.

Both tDRs derived from tRNA–Ser–AGA were also mostly associated with 40S subunits: 79% (northern blot) or 80% (qRT-PCR) of tDR-62:82-Ser–AGA and 78% (northern blot) or 89% (qRT-PCR) of tDR-58:82-Ser–AGA. However, a prominent amount of tRNA–Ser-derived tDRs were also co-migrating with 60S subunits: 14% (northern blot and qRT-PCR) of tDR-62:82-Ser–AGA and 19% (northern blot) or 18% (qRT-PCR) of tDR-58:82-Ser–AGA. Ser–tDRs were detected in the monosome (up to 6%) and polysome (1%) fractions.

Such results suggest that high concentrations of tDRs interfere with translation and ribosome function, shifting their association away from active polysomes and toward subunits (especially 40S), which likely reflects a stress response or inhibitory role.

While some differences were noted in the detection of Ser–tDRs in monosome and polysome fractions, the overall patterns of tDR association with ribosomal particles observed using northern blotting and qRT-PCR are largely consistent with those obtained using the tDR-quant method.. An important advantage of tDR-quant is its ability to detect very low amounts of tDRs through sensitive scintillation counting, which is particularly useful in quantifying ribosomal associations that might be below the detection threshold of standard techniques. While northern blotting and qRT-PCR can certainly provide detailed insights into tDR–ribosome interactions, especially when experimental conditions such as electroporation are used to modulate intracellular tDR levels, tDR-quant offers the added benefit of distinguishing between endogenous and radiolabeled tDRs. This distinction is particularly valuable when studying dynamic changes in tDR behavior under varying cellular conditions, such as stress, where changes in tDR abundance can influence ribosomal association (Bąkowska-Żywicka et al. 2016b). To further validate the tDR-quant results obtained under varying tDR abundance, we have introduced multiple moderate, temporary stress conditions to yeast cells and assessed tDR/ribosomal particles interactions with northern blots performed on yeast extracts derived from stressed cells, to corroborate the tDR-quant results obtained with different amounts of tDRs. Induction of stress conditions on yeast spheroplasts was not possible, since these cells are deprived of the cell wall, making them more sensitive to nonoptimal conditions and therefore not reflecting the true status of stress response.

**Figure 5.**
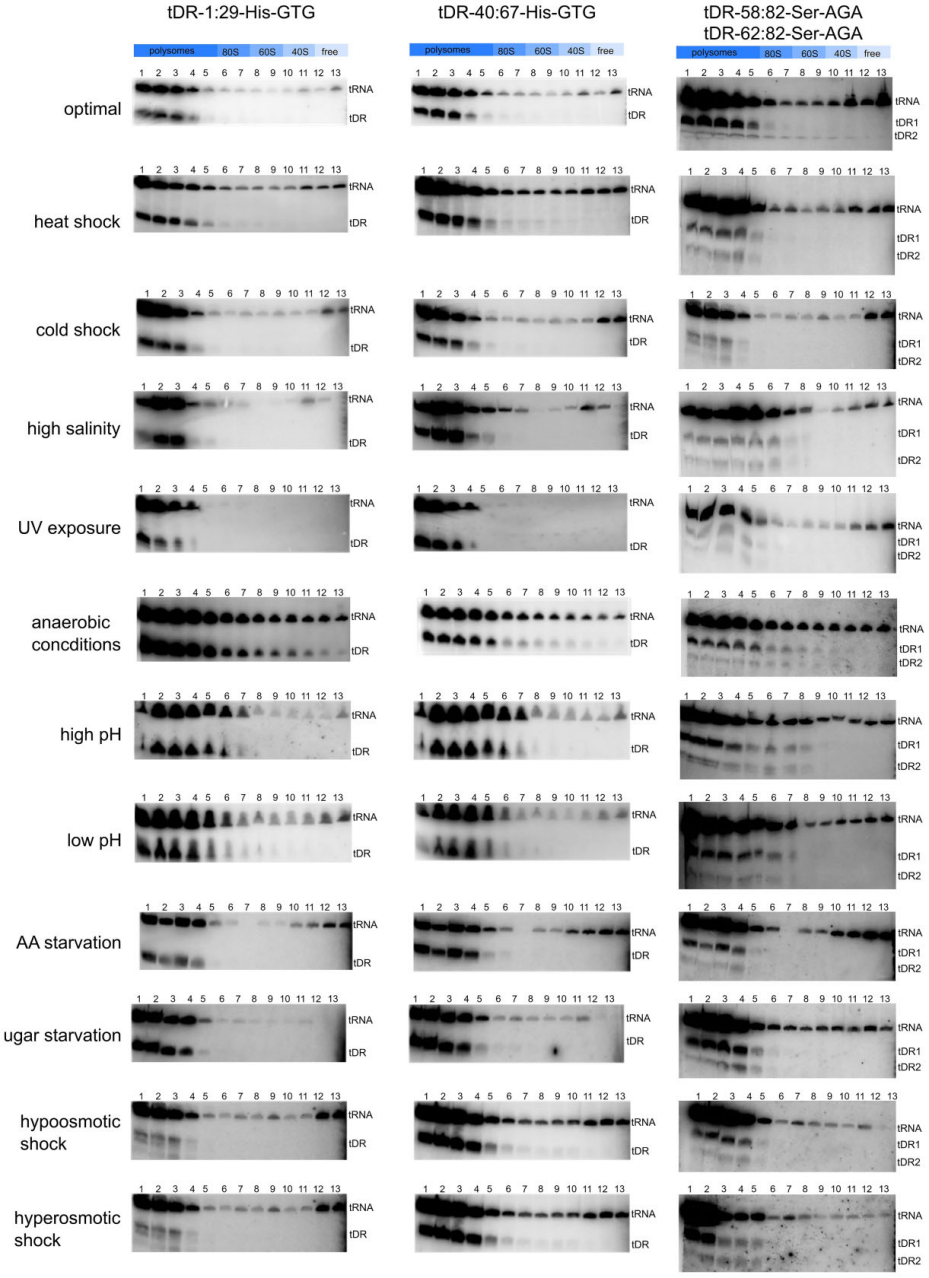
Representative northern blot analyses corresponding to quantification data shown in Figs. 6–9. Numbers correspond to fractions from polysome profiles. The position of bands corresponding to full length tRNA and tDRs are presented.

**Figure 6.**
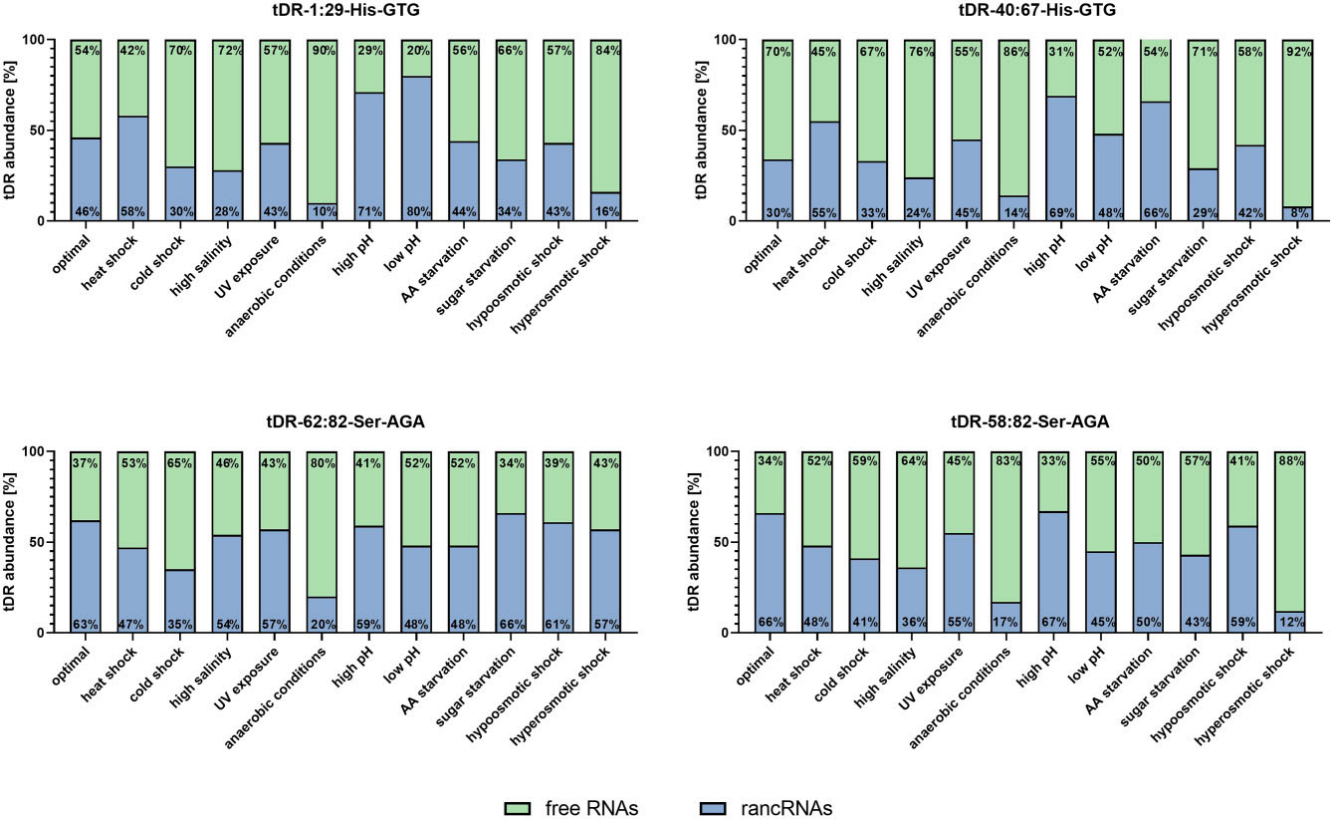
Stress-dependent changes in the ribosome association of tDRs. Northern blot analysis showing the proportion of selected tDRs associated with ribosomal particles (rancRNAs) under various stress conditions.

First, we have inspected whether the association of tDRs with ribosomal particles changes during stress, that is, how much of tDR present in the cell during particular stress conditions is associated with the ribosomal particles (is rancRNA). We observed significant changes in the proportion of ribosome-associated *versus* nonassociated tDRs derived from both tRNA–His and tRNA–Ser during multiple stress conditions, as revealed by northern blots (Figs 5 and 6). During optimal conditions, 46% of tDR-1:29-His–GTG, 30% of tDR-40:67-His–GTG, 63% of tDR-62:82-Ser–AGA, and 66% of tDR-58:82-Ser–AGA were present as rancRNAs. The least tDRs were co-migrating with ribosomal particles during anaerobic conditions: 10% of tDR-1:29-His–GTG (36% less than in optimal conditions), 14% of tDR-40:67-His–GTG (16% less than in optimal conditions), 20% of tDR-62:82-Ser–AGA (43% less than in optimal conditions), and 17% of tDR-58:82-Ser–AGA (49% less than in optimal conditions). The most tDRs were bound to the ribosomes during low pH conditions conditions: 80% of tDR-1:29-His–GTG (34% more than in optimal conditions), high pH conditions: 69% of tDR-40:67-His–GTG (39% more than in optimal conditions), and 67% of tDR-58:82-Ser–AGA (1% increase), and during sugar starvation, when 66% of tDR-62:82-Ser–AGA (3% increase). A larger, than under optimal conditions, pool of tDR-1:29-His–GTG was present as rancRNA under heat shock, high, and low pH. For tDR-40:67-His–GTG, the stress conditions that resulted in higher rancRNA-tDR pool than under optimal conditions were: heat or cold shock, UV exposure, high or low pH, amino acids starvation, and hypoosmotic shock. Most stresses caused a decrease in the pool of tRNA–Ser-derived ranc–tDRs.

**Figure 7.**
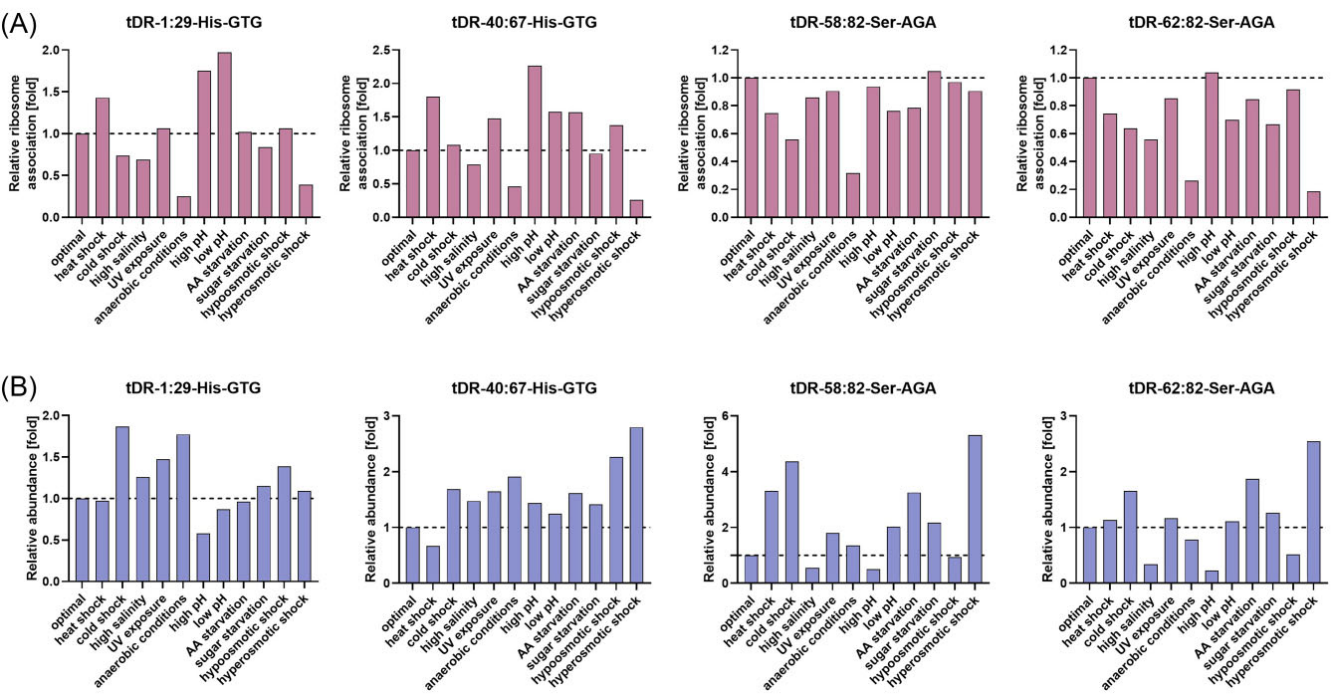
Stress-dependent tDR/ribosome association and tDR abundance in yeast cells. (A) Quantitation of band intensity on northern blot corresponding to tDRs in rancRNAs fractions of polysome profiles derived from yeast subjected to 11 environmental conditions in relation to control conditions. (B) Quantitation of band intensity on northern blot corresponding to tDRs total RNA derived from yeast subjected to 11 environmental conditions in relation to control conditions. Data in (B) are based on results presented in Bąkowska-Żywicka et al. (2016b). The reference value in optimal conditions was set to 1.

In order to reveal if deregulated stress-dependent tDR/ribosome association might be the consequence of stress-dependent tDRs abundance in yeast cells, we compared the relative tDR/ribosome association profiles (Fig. 7A) with previously published by our group tDR abundance profiles [Fig. 7B, based on results presented in Bąkowska-Żywicka et al. (2016b)]. We observed that both, tRNA–His and tRNA–Ser-derived tDRs associate with ribosomal particles, as well as their abundance in yeast is stress-dependent. However, we did not observe a clear correlation between the amount of particular tDR and its association with the ribosomes. Such a situation suggests that increased tDR/ribosome association during particular stress is not a result of stress-related increased tDR abundance. As an example, tDR-1:29-His–GTG co-migration with ribosomal particles during high pH stress was 1.8-fold higher than in optimal conditions (Fig. 7A), but its abundance during this stress was 0.6-fold lower than in optimal growth (Fig. 7B). On the other hand, the same tDR, although present in yeast cells in larger amounts during anaerobic growth (1.7-fold higher than in optimal conditions), was interacting with ribosomal particles to lesser extent than in optimal conditions (0.3-fold lower than in optimal growth).

**Figure 8.**
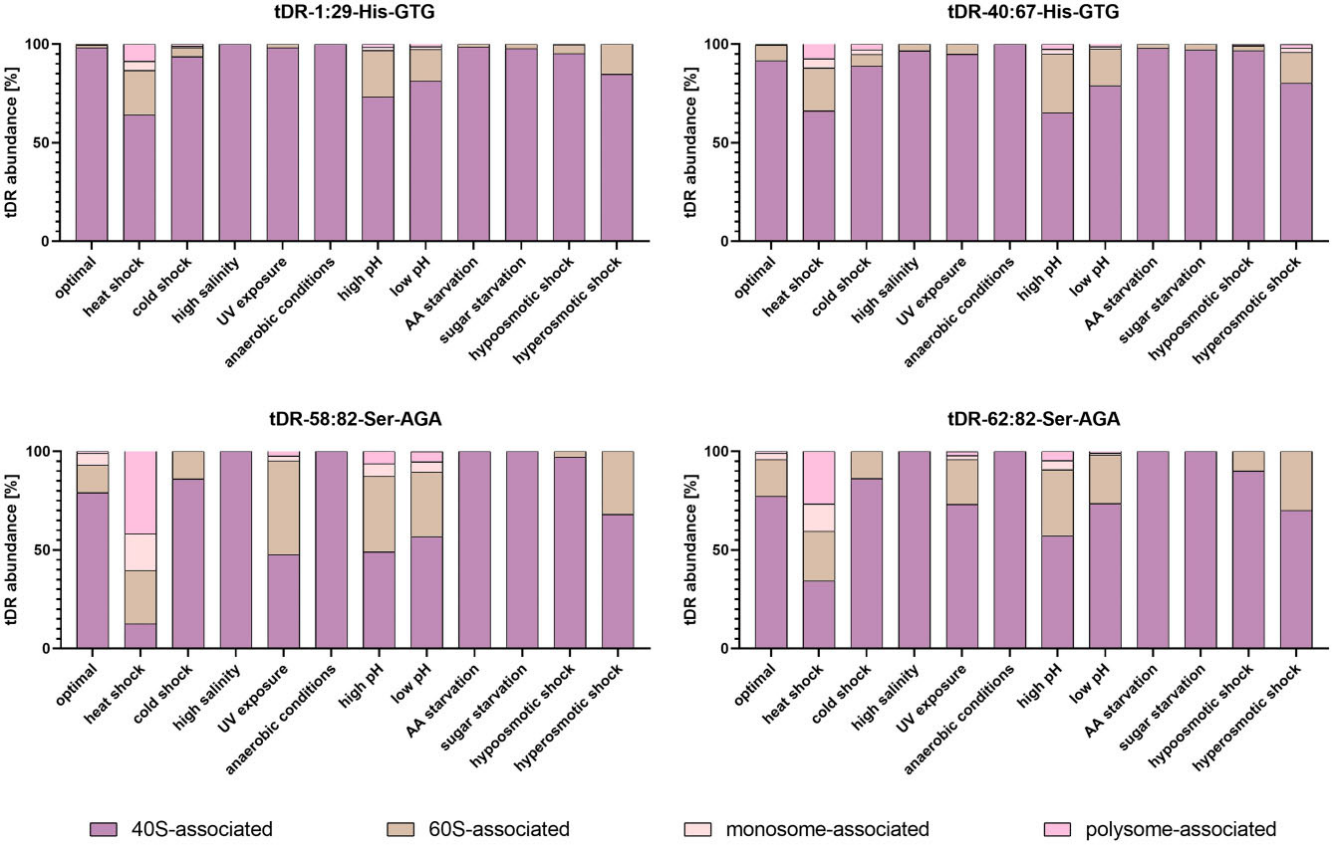
The ratio of co-migration of particular tDRs with ribosomal particles. Quantitation of band intensity on northern blot corresponding to tDR in 40S, 60S, monosomes, and polysomes fractions is presented.

In the next step, we investigated the influence of stress conditions on tDRs’ association with particular ribosomal particles. We observed that both tRNA–His-derived tDRs were primarily co-migrating with small ribosomal 40S subunits in all stress conditions (Fig. 8). A total of 64% of tDR-1:29-His–GTG and 66% of tDR-40:67-His–GTG were co-migrating with 40S during heat shock (and this is the stress condition where the lowest percentage of ranc–tDRs–His were associating with the small ribosomal subunits), 100% of both tDRs–His–GTG were co-migrating with 40S during anaerobic conditions, and during high salinity all ranc–tDR-1:29-His–GTG was associating with 40S. Such a situation was also observed for tDRs–Ser: the lowest association with 40S was observed under heat shock (12% of tDR-62:82-Ser–AGA and 34% of tDR-58:82-Ser–AGA were bound to 40S), and the highest—under anaerobic and high pH conditions (100% of both tDRs–Ser were associated with 40S).

Visible amounts of tDRs–His were also co-migrating with large ribosomal 60S subunits under heat shock, high or low pH, and hyperosmotic shock. During these stresses tDRs–Ser were also co-migrating with 60S subunits, but additionally during cold shock, UV exposure, and hypoosmotic shock, as well as under optimal conditions. Prominent amounts of tDRs–Ser were co-migrating with the monosomes and the polysomes during heat shock.

**Figure 9.**
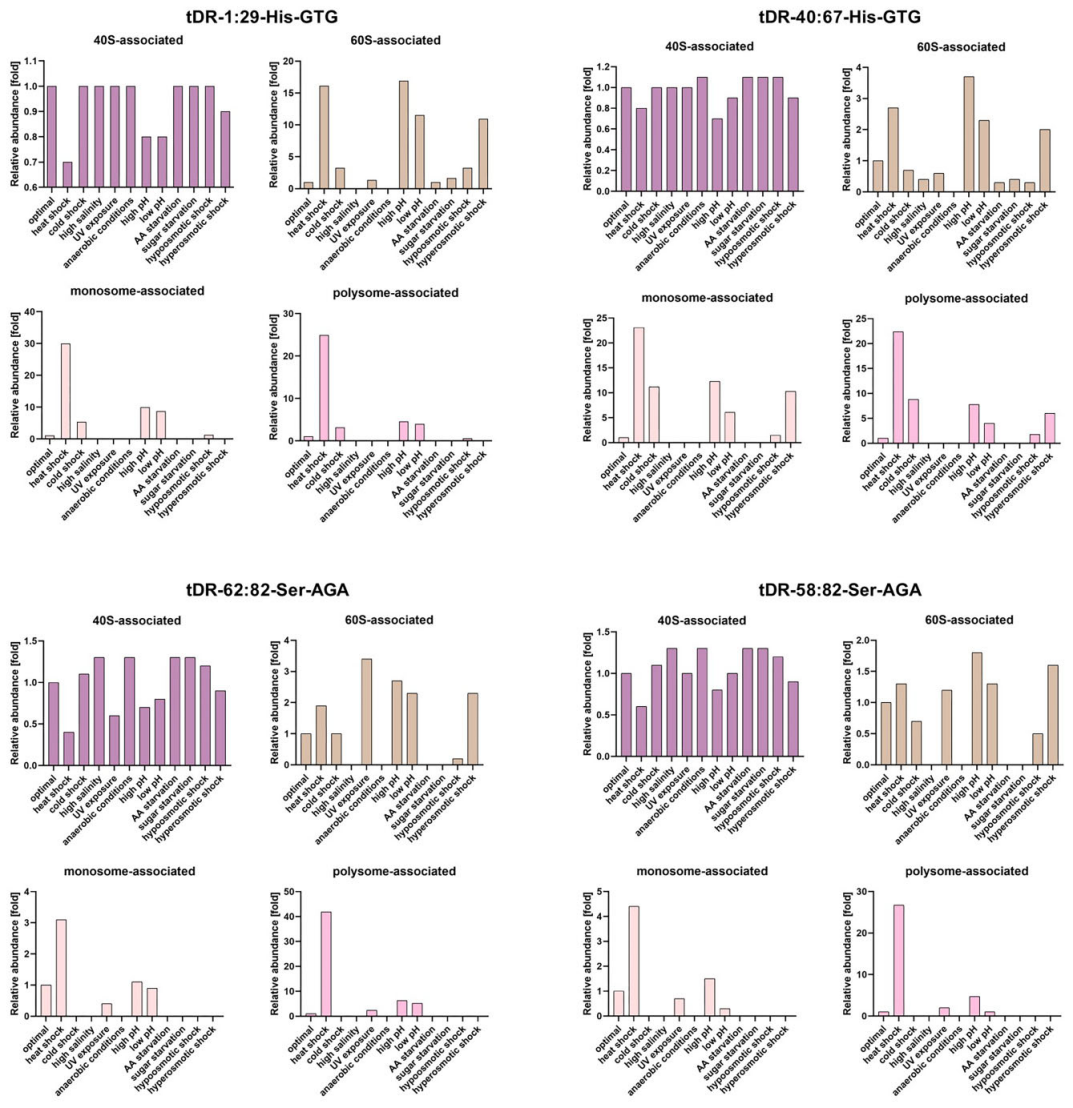
Stress-dependent tDR/ribosomal particles association in yeast cells. Quantitation of band intensity on northern blot corresponding to tDRs in 40S-associated, 60S-associated, monosome-associated, and polysome-associated RNA fractions from polysome profiles derived from yeast subjected to 11 environmental conditions in relation to control conditions. The reference value in optimal conditions was set to 1.

Comparing the tDR/ribosomal particles association profiles obtained under stress with the profile captured during optimal conditions, it appeared that the association of both tDRs derived from tRNA–His with 40S ribosomal subunits was diminished under heat shock, high or low pH conditions, and during hyperosmotic shock (Fig. 9). Under these stress conditions, significantly higher association of tDR-1:29-His–GTG with 60S subunits, monosomes, and polysomes (but not under hyperosmotic shock) was observed. tDR-40:67-His–GTG associated with 60S subunits, monosomes, as well as polysomes at significantly higher degree than under optimal conditions during stresses that caused its diminished co-migration with 40S subunits, but also under cold shock (monosome and polysome association). The association of both tDRs derived from tRNA–Ser with 40S ribosomal subunits was diminished under heat shock, high or low pH conditions, and during hyperosmotic shock, like for tDRs–His, but also under UV exposure. Under these stress conditions, a significantly higher association of both tDRs–Ser with 60S subunits was observed. Additionally, both tDRs–His co-migrated with the monosomes and the polysomes under heat shock and high pH significantly more than under optimal conditions, and tDR-62–82-Ser–AGA—also under low pH conditions.

## Discussion

This study provides a detailed analysis of how four selected tDRs interact with yeast ribosomes, offering new insights into their potential functional roles.The use of various techniques, including polysome profiling, tDR-quant, northern blotting, and qRT-PCR, allowed for a comprehensive analysis of tDR behavior under different conditions. While northern blotting provided snapshots of tDR abundance under specific stress conditions, tDR-quant offers a dynamic view of tDR interactions with ribosomal particles, particularly under different tDR doses. Our approach provides a sensitive means to trace radiolabeled, electroporated tDRs and assess their interactions with ribosomal particles under varying conditions, offering a complementary perspective to existing methods for studying endogenous tDRs.

The observation that spheroplasting and electroporation did not significantly alter the polysome profiles of yeast cells is consistent with findings from other studies. A study on *S. cerevisiae* reported that osmotic stress leads to transient inhibition of translation initiation; however, the polysome profiles remained largely unaffected during the stress response (Uesono and Toh-E 2002). Moreover, in 2020, David Tollervey group revealed that under heat shock and glucose starvation conditions, scanning initiation factors such as eIF4A, eIF4B, and Ded1 dissociate from the 5′ end of mRNAs, leading to a rapid inhibition of translation initiation, but this inhibition did not result in significant changes to polysome profiles (Bresson et al. 2020). A similar situation was observed in fission yeast under UV light exposure (Knutsen et al. 2015). All these data, together with our results, suggest that polysome integrity can be maintained even under stress conditions.

Despite unaltered polysome profiles, our study demonstrated a dose-dependent decrease in translation efficiency upon increasing tDR concentrations, highlighting the complex regulatory roles of tDRs beyond mere ribosome occupancy. Several studies are pointing to the dose-dependent regulatory roles of tDRs in translation control, particularly under stress conditions. Our group previously showed that in *S. cerevisiae*, both 5′- and 3′-tDRs bind directly to the ribosomes in a stress-dependent manner, and this binding correlates with inhibition of protein biosynthesis under specific environmental conditions (Bąkowska-Żywicka et al. 2016a). Moreover, we observed a dose-dependence of yeast tDR influence on translation (Mleczko et al. 2018). A study in the halophilic archaeon *Haloferax volcanii* identified a tDR-Val, highly accumulated under alkaline stress conditions, which binds to the small ribosomal subunit, displacing mRNA from the initiation complex, and resulting in global translation attenuation in a dose-dependent manner (Gebetsberger et al. 2017). Also, a breakthrough study by Pavel Ivanov identified human angiogenin-induced tDRs that are potent to inhibit translation initiation through displacing initiation factors from mRNA in a dose-dependent manner, and their action is thought to be part of a broader stress response mechanism (Ivanov et al. 2011).

It is crucial to underscore that the newly established tDR-quant method revealed that tDRs derived from tRNA–His–GTG predominantly associate with 40S subunits, while tDRs from tRNA–Ser–AGA show a broader distribution, including 60S subunits and 80S monosomes. This differential association may reflect distinct functional roles or regulatory mechanisms associated with specific tDRs. Notably, the association of tDRs with ribosomal subunits was influenced by stress conditions, with varying degrees of co-migration observed under different stressors. For example, during heat shock, both tDRs derived from tRNA–His–GTG were primarily associated with 40S subunits, whereas under anaerobic conditions, they were entirely associated with 40S subunits. The observed changes in tDR association with ribosomal subunits under various stress conditions suggest that tDRs play adaptive roles in translation regulation during stress responses. These findings align with research indicating that cells can reprogram translation in response to stress, favoring the translation of short, efficiently translated mRNAs (Gaikwad et al. 2021).

While the tDR-quant method provides a powerful and sensitive approach for assessing tDR interactions with ribosomal particles, several limitations must be considered. First, the artificial introduction of radiolabeled synthetic tDRs via electroporation into yeast spheroplasts may not fully recapitulate the endogenous behavior, processing, or modification status of native tDRs, particularly at higher concentrations where nonphysiological interactions may occur. Second, the use of spheroplasts, although preserving polysome integrity, limits the applicability of the method to natural stress responses due to their increased sensitivity and inability to sustain prolonged environmental perturbations. Additionally, tDR-quant detects only exogenous, labeled tDRs, precluding the analysis of endogenous small RNA competition or the broader cellular small RNA landscape. Finally, the technique involves radioactive labeling. Despite these constraints, tDR-quant remains a valuable tool for controlled and quantitative studies of ribosome–tDR interactions.

## Conclusions

This study accentuates the complexity of tDR functions in translation regulation in yeast *S. cerevisiae*. The dose-dependent effects on translation efficiency and the differential association with ribosomal subunits highlight the nuanced roles of tDRs in cellular adaptation to stress. These insights contribute to a deeper understanding of post-transcriptional regulation and the adaptive mechanisms employed by yeast cells in response to environmental challenges.
